# Perspective of Medical Students on the COVID-19 Pandemic: Survey of Nine Medical Schools in Uganda

**DOI:** 10.2196/19847

**Published:** 2020-06-19

**Authors:** Ronald Olum, Jonathan Kajjimu, Andrew Marvin Kanyike, Gaudencia Chekwech, Godfrey Wekha, Dianah Rhoda Nassozi, Juliet Kemigisa, Paul Mulyamboga, Oscar Kabagambe Muhoozi, Lauryn Nsenga, Musilim Lyavala, Asaph Asiimwe, Felix Bongomin

**Affiliations:** 1 School of Medicine College of Health Sciences Makerere University Kampala Uganda; 2 Faculty of Medicine Mbarara University of Science and Technology Mbarara Uganda; 3 Faculty of Health Sciences Busitema University Mbale Uganda; 4 School of Health Sciences College of Health Sciences Makerere University Kampala Uganda; 5 Faculty of Medicine Gulu University Gulu Uganda; 6 Faculty of Biology, Medicine and Health King Caesar University Kampala Uganda; 7 Faculty of Clinical Medicine and Dentistry Kampala International University Ishaka-Bushenyi Uganda; 8 School of Medicine Kabale University Kabale Uganda; 9 Faculty of Medicine Islamic University in Uganda Mbale Uganda; 10 School of Health Sciences Soroti University Soroti Uganda; 11 Department of Medicine College of Health Sciences Makerere University Kampala Uganda; 12 Department of Medical Microbiology and Immunology Faculty of Medicine Gulu University Gulu Uganda

**Keywords:** knowledge, attitude, practices, COVID-19, medical students, Uganda, medical education, perspective

## Abstract

**Background:**

The coronavirus disease (COVID-19) pandemic is a global public health concern affecting over 5 million people and posing a great burden on health care systems worldwide.

**Objective:**

The aim of this study is to determine the knowledge, attitude, and practices of medical students in Uganda on the COVID-19 pandemic.

**Methods:**

We conducted an online, descriptive cross-sectional study in mid-April 2020, using WhatsApp Messenger. Medical students in 9 of the 10 medical schools in Uganda were approached through convenience sampling. Bloom’s cut-off of 80% was used to determine good knowledge (≥12 out of 15), positive attitude (≥20 out of 25), and good practice (≥12 out of 15).

**Results:**

The data of 741 first- to fifth-year medical students, consisting of 468 (63%) males with a mean age of 24 (SD 4) years, were analyzed. The majority (n=626, 84%) were pursuing Bachelor of Medicine and Bachelor of Surgery degrees. Overall, 671 (91%) had good knowledge, 550 (74%) had a positive attitude, and 426 (57%) had good practices. Knowledge was associated with the 4th year of study (adjusted odds ratio [aOR] 4.1, 95% CI 1.6-10.3; *P*<.001). Attitude was associated with the female sex (aOR 0.7, 95% CI 0.5-1; *P*=.04) and TV or radio shows (aOR 1.1, 95% CI 0.6-2.1; *P*=.01). Practices were associated with the ≥24 years age category (aOR 1.5, 95% CI 1.1-2.1; *P*=.02) and online courses (aOR 1.8, 95% CI 1.1-3.2; *P*=.03). In total, 592 (80%) medical students were willing to participate in frontline care if called upon.

**Conclusions:**

Medical students in Uganda have sufficient knowledge of COVID-19 and will be a large reservoir for health care response when the need arises.

## Introduction

In late December 2019, a pneumonia of unknown cause was first reported in Wuhan City, China [1]. The World Health Organization (WHO) later named the disease the coronavirus disease (COVID-19). COVID-19 caused by the novel coronavirus, also known as severe acute respiratory syndrome coronavirus 2 was linked to a seafood and wild animal wholesale market in Wuhan, Hubei Province, China [2]. COVID-19 has since rapidly spread across the world with multiple countries and was declared a global pandemic on March 11, 2020, by the WHO [3].

Over 5.5 million cases and 350,000 deaths have been reported worldwide [4]. Over 30% of the confirmed cases and 25% of COVID-19 deaths worldwide are in the United States alone [5]. As of May 27, 2020, Africa has over 83,000 confirmed cases and 2000 deaths [5]. The strategies established worldwide to reduce the transmission are mostly behavioral (eg, social distancing, regular washing of hands), largely depending on rapid change in behavior, which relies on one’s knowledge about the problem, ability to perceive the risk, and willingness to change their attitude [6]. So far, over 10,000 health care workers have been infected with the virus and over 100 have died from COVID-19 [7]. In countries with large amounts of COVID-19 cases such as Italy, the United States, and the United Kingdom, final year medical students and foundation year doctors were fast-tracked into the next level of their career with expedited assessment to help the severely overwhelmed health workforce [8,9]. Empowering medical students with adequate knowledge will place them at the forefront of health education to give the public correct information and refute myths and false information about COVID- 19 [10].

A recent study among Iranian medical students spending their clinical courses in university teaching hospitals all over Iran, found a significantly negative correlation between self-reported preventive behaviors and risk perception, which is needed to reduce stress, anxiety, and risk perception, which are the major problems in disease outbreaks [11]. To our knowledge, no study has been published assessing the knowledge, attitude, and practices (KAP) of medical students in Uganda and Africa at large toward COVID-19, necessitating this study. We, therefore, aimed to assess the KAP of medical students in Uganda toward the COVID-19 pandemic.

## Methods

### Study Design

We conducted an online, descriptive cross-sectional study between Monday, April 13 and Sunday, April 19, 2020. A quantitative analysis approached was used.

### Study Settings

There are 10 universities in Uganda offering undergraduate medical degrees, namely, Makerere University (Mak), Mbarara University of Science and Technology (MUST), Gulu University (GU), Kampala International University (KIU), Kabale University (KU), Busitema University (BU), Islamic University in Uganda, Soroti University (SU), King Caesar International University, and Uganda Christian University (UCU). Mak, GU, MUST, BU, KU, and SU are public universities, and the remaining universities are private. UCU was not included in this survey because of a lack of a representative. The combined population size of all these medical schools is about 6000-8000 students.

### Study Population

Medical students pursuing the following undergraduate degree programs in various universities were targeted: Bachelor of Medicine and Bachelor of Surgery (MBChB), Bachelor of Dental Surgery (BDS), Bachelor of Nursing (BNUR), and Bachelor of Pharmacy (BPHARM).

### Inclusion and Exclusion Criteria

Individuals 18 years or older were included in the study after an informed consent was obtained. Students who were too ill to participate were excluded.

### Sampling Procedure and Data Collection

At the time of data collection, Uganda was in a total lockdown; all schools, universities, and institutions were closed. Therefore, we opted to use WhatsApp Messenger (Facebook Inc) for enrolling potential participants. By employing a convenience sampling method, we identified all the existing WhatsApp groups of medical students in the various universities. The Google Form link to the questionnaire was sent to the enrolled participants via the identified WhatsApp groups with approximately 2500 students.

### Study Variables

Independent variables were the demographic characteristics sex, age, education institution, and sources of information on COVID-19, and dependent variables were knowledge, attitude, and practices toward COVID-19.

Bloom’s cut-off of 80% was used to determine whether a medical student had good knowledge, positive attitude, and good practice or not [12].

*Knowledge* was assessed using a 12-item questionnaire adapted from Zhong et al [13] and modified to suit medical students, each correct answer weighing one point. The questions were about clinical presentations, transmission, prevention, and control of COVID-19. Each correct response was weighted as 1 point and 0 for incorrect responses. The total score was 15, and ≥12 (ie, 80%) correct responses was considered good knowledge.

*Attitudes* were assessed using 5 Likert-item questions that have been adopted from Goni et al [14] and modified appropriately for COVID-19 by the authors. The responses were strongly disagree, disagree, neutral, agree, and strongly agree, each weighing 1-5 for each positive statement. Some questions were reversed to eliminate biases of giving a single similar response in all the items. The total score was 25, and ≥20 (ie, 80%) correct responses was considered a positive attitude.

*Practices* were assessed using 5 Likert-item questions that have been developed from the WHO and Ministry of Health Uganda recommended practices for prevention of COVID-19 transmission (ie, hand washing, avoiding crowded places, keeping social distance [1 meter apart], avoiding touching of face, and avoiding handshakes). The responses were always, occasional, and never, each weighing 3, 2, and 1 point for a good practice. The total score was 15, and ≥12 (ie, 80%) correct responses were considered good practices.

The questionnaire can be accessed in Multimedia Appendix 1.

### Data Management and Analyses

Fully completed questionnaires were extracted from Google Forms and exported to Microsoft Excel 2016 (Microsoft Corporation) for cleaning and coding. The cleaned data was exported to Stata (StataCorp) version 15.1 for analyses. Numerical data was summarized as means and standard deviations. Categorical data was summarized as frequencies and proportions. Associations between independent variables and dependent variables were assessed using chi-square test and multivariate analysis in Stata 15.1 software. A *P*<.05 is considered statistically significant.

### Ethical Consideration

The study was cleared by Mulago Hospital Research Ethics Committee, protocol number MHREC 1866. All participants consented to the study, and it was conducted according to the *Declaration of Helsinki.*

## Results

### Sociodemographic Characteristics of the Participants

Overall, 806 participants responded to the study. After cleaning and validating the data, 741 valid responses were exported for analysis. The vast majority of the participants were male (n=468, 63%) and pursuing MBChB degree (n=626, 84%). Up to 24% (n=177) were from Makerere University College of Health Sciences, the oldest medical school in Uganda. The majority of the participants used mass media like televisions and social media to access information on COVID-19 (79% vs 76%, respectively). Only 2% (n=18) of the participants were from Soroti University School of Health Sciences, the youngest medical school in Uganda. Table 1 summarizes the characteristics of participants.

### Knowledge of Medical Students on COVID-19

The majority of medical students identified fever, cough, and difficulty in breathing as the main clinical symptoms of COVID-19 (95%, 85%, and 88%, respectively). However, only 19% knew that myalgia was a main clinical symptom of COVID-19 (Table 2).

The mean knowledge score of the participants was 13.1 (SD 1.2) indicating a good overall knowledge among medical students. The vast number of the medical students had sufficient knowledge (score≥12, n=671/741, 91%) on COVID-19 main clinical symptoms, transmission, and prevention. Table 3 summarizes the mean knowledge score of participants.

**Table 1:** Sociodemographic characteristics of the participants (N=741).

Variables		Participants
**Sex, n (%)**		
	Male	468 (63)
	Female	273 (37)
**Age (years), mean (SD)**		24.0 (4)
	18-23, n (%)	425 (57)
	≥24, n (%)	316 (43)
**University, n (%)**		
	Busitema University	94 (13)
	Gulu University	67 (9)
	Islamic University in Uganda	128 (17)
	Kabale University	88 (12)
	Kampala International University	76 (10)
	King Caesar University	29 (4)
	Makerere University	177 (24)
	Mbarara University of Science and Technology	64 (9)
	Soroti University	18 (2)
**Program, n (%)**		
	Bachelor of Medicine and Bachelor of Surgery	626 (84)
	Bachelor of Dental Surgery	20 (3)
	Bachelor of Nursing	63 (9)
	Bachelor of Pharmacy	32 (4)
**Year of study, n (%)**		
	1st	109 (15)
	2nd	150 (20)
	3rd	168 (23)
	4th	221 (30)
	5th	93 (13)
**Source of information on the coronavirus disease, n (%)**		
	Webinar	107 (14)
	TV or radio	583 (79)
	Journal and articles	292 (39)
	Social media	565 (76)
	Websites	354 (48)
	Online courses	77 (10)

**Table 2:** Responses of Ugandan medical students (N=741) to questions on knowledge about COVID-19.

Question		Response, n (%)	
		True	False
SARS-COV-2^a^ the virus that cause COVID-19^b^ is a DNA virus (*false*)		240 (32)	501 (68)
**The main clinical symptoms of COVID-19 are (tick all that apply)**			
	Cough (*true*)	631 (85)	110 (15)
	Fever *(true*)	703 (95)	38 (5)
	Myalgia (*true*)	143 (19)	598 (81)
	Dyspnea (*true*)	649 (88)	92 (12)
	Sore throat	551 (74)	190 (26)
	Runny nose	358 (48)	383 (52)
	Headache	258 (35)	483 (65)
	Sneezing	546 (74)	195 (26)
	Confusion	13 (2)	728 (98)
	Diarrhea	67 (9)	674 (91)
There is currently no effective cure for COVID-19, but early symptomatic and supportive treatment can help most patients recover from the infection (*true*)		738 (100)	3 (0)
Not all persons with COVID-19 will develop severe cases. Only those who are elderly, have chronic illnesses, and are obese are more likely to be severe cases (*true*)		669 (90)	72 (10)
Persons with COVID-2019 cannot transmit the virus to others when a fever is not present (*false*)		23 (3)	718 (97)
The COVID-19 virus spreads via respiratory droplets of infected individuals. (*true*)		734 (99)	7 (1)
SARS-COV-2 the virus that causes COVID-19 cannot persist on surfaces of objects for hours (*false*)		74 (10)	667 (90)
Wearing general medical masks can prevent one from acquiring infection by the COVID-19 virus (*true*)		642 (87)	99 (13)
It is not necessary for children and young adults to take measures to prevent the infection by the COVID-19 virus (*false*)		22 (3)	719 (97)
To prevent the infection by COVID-19, individuals should avoid going to crowded places such as bus parks and avoid taking public transportations (*true*)		735 (99)	6 (1)
Isolation and treatment of people who are infected with the COVID-19 virus are effective ways to reduce the spread of the virus (*true*)		739 (100)	2 (0)
People who have contact with someone infected with the COVID-19 virus should be immediately isolated in a proper place. In general, the observation period is 14 days (*true*)		738 (100)	3 (0)

^a^SARS-CoV-2: severe acute respiratory syndrome coronavirus 2.

^b^COVID-19: coronavirus disease.

Age, year of study, and source of information were significant predictors of knowledge on bivariate analysis, however, they lost significance in the multivariate analysis. Medical students who used journals or articles (*P*=.03) and websites (*P*=.03) as a source of information significantly had sufficient knowledge than others (Table 3). On multivariate analysis, fourth year medical students in Uganda significantly had more sufficient knowledge than their first year counterparts (adjusted odds ratio [aOR] 4.1, 95% CI 1.6-10.3, *P*<.01). Age, sex, university, program, and source of information on COVID-19 were not statistically significant in the multivariate analysis (Table 4).

**Table 3:** Mean scores and chi-square test showing knowledge, attitude, and practices of medical students in Uganda toward COVID-19.

Variables (N=741)			Knowledge						Attitude						Practice				
			Mean (SD)		Sufficient, n (%)		*P* value		Mean (SD)		Positive, n (%)		*P* value		Mean (SD)		Good, n (%)		*P* value
Overall			13.1 (1.2)		671 (91)		N/A^a^		20.8 (3.2)		550 (74)		N/A		11.8 (1.9)		426 (57)		N/A
**Sex**							.06						.04						.99
	Male	13.2 (1.1)		431 (92)				21 (3.3)		359 (77)				11.8 (2)		269 (57)			
	Female	13 (1.3)		240 (88)				20.4 (3)		191 (70)				11.8 (1.7)		157 (58)			
**Age (years)**							.046						.55						.01
	18-23	13 (1.2)		377 (89)				20.9 (2.7)		319 (75)				11.7 (1.8)		228 (54)			
	≥24	13.3 (1.2)		294 (93)				20.6 (3.8)		231 (73)				12 (1.9)		198 (63)			
**University**							.30						.20						.05
	Busitema University	13.3 (1.1)		89 (95)				20.9 (3.6)		68 (72)				11.7 (1.8)		54 (57)			
	Gulu University	13.1 (1.2)		59 (88)				20.7 (3.6)		52 (78)				11.4 (1.9)		32 (48)			
	Islamic University in Uganda	12.9 (1.3)		109 (85)				20.6 (2.8)		94 (73)				11.9 (2)		76 (59)			
	Kabale University	13.1 (1.3)		78 (89)				21.7 (2.2)		73 (83)				12.1 (1.5)		58 (66)			
	Kampala International University	13.1 (1)		70 (92)				21 (3.1)		61 (80)				12.5 (1.7)		53 (70)			
	King Caesar University	13.2 (1)		27 (93)				20.9 (2.5)		24 (83)				12.2 (1.6)		19 (66)			
	Makerere University	13.2 (1.2)		165 (93)				20.2 (3.6)		120 (68)				11.5 (2.1)		88 (50)			
	Mbarara University of Science and Technology	13.1 (1.2)		57 (89)				20.8 (3.2)		45 (70)				11.8 (1.7)		35 (55)			
	Soroti University	13.2 (1.2)		17 (94)				21 (2.6)		13 (72)				12.2 (1.9)		11 (61)			
**Program**							.11						.04						.06
	Bachelor of Medicine and Bachelor of Surgery	13.2 (1.1)		574 (92)				20.9 (3)		476 (76)				11.8 (1.8)		354 (57)			
	Bachelor of Dental Surgery	12.8 (1.4)		17 (85)				19.3 (5.2)		11 (55)				11.1 (2.9)		9 (45)			
	Bachelor of Nursing	13 (1.7)		53 (84)				20.4 (4.2)		41 (65)				11.9 (1.8)		38 (60)			
	Bachelor of Pharmacy	12.9 (1.4)		27 (84)				20.5 (3.7)		22 (69)				12.5 (1.9)		25 (78)			
**Year of study**							<.001						.78						.98
	1st	12.8 (1.3)		91 (83)				20.6 (3)		83 (76)				11.8 (1.9)		63 (58)			
	2nd	12.8 (1.3)		131 (87)				20.9 (2.9)		112 (75)				11.8 (1.9)		87 (58)			
	3rd	13.1 (1.1)		150 (89)				20.9 (2.8)		122 (73)				11.8 (1.8)		93 (55)			
	4th	13.4 (1.1)		212 (96)				20.9 (3.2)		168 (76)				11.9 (1.8)		128 (58)			
	5th	13.3 (1.1)		87 (94)				20.3 (4.3)		65 (70)				11.8 (2.1)		55 (59)			
**Source of information on COVID-19^b^**																			
	Webinar	13 (1.3)		96 (90)		.75		20.7 (3.7)		77 (72)		.56		12.1 (2.3)		71 (66)		.04	
	TV or radio	13.2 (1.1)		531 (91)		.35		20.9 (3.1)		445 (76)		.01		11.8 (1.9)		332 (57)		.57	
	Journal and articles	13.2 (1.1)		273 (93)		.03		20.9 (2.9)		226 (77)		.11		12.1 (1.8)		185 (63)		.01	
	Social media	13.2 (1.2)		518 (92)		.06		20.9 (3.1)		425 (75)		.27		11.8 (1.8)		327 (58)		.70	
	Websites	13.2 (1.1)		329 (93)		.03		20.7 (3.1)		261 (74)		.77		11.8 (1.8)		216 (61)		.06	
	Online courses	13.2 (1.2)		73 (95)		.18		20.9 (3.4)		58 (75)		.82		12.4 (1.8)		56 (73)		<.001	

^a^Not applicable.

^b^COVID-19: coronavirus disease.

**Table 4:** Multivariate analysis showing factors associated with knowledge, attitude, and practices toward COVID-19 among Ugandan medical students.

Variable		Knowledge		Attitude		Practices	
		aOR^a^ (95% CI)	*P* value	aOR (95% CI)	*P* value	aOR (95% CI)	*P* value
**Sex**							
	Male	1	N/A^b^	1	N/A	1	N/A
	Female	0.7 (0.4-1.2)	.18	0.7 (0.5-1)	.04	1.1 (0.8-1.5)	.61
**Age (years)**							
	18-23	1	N/A	1	N/A	1	N/A
	≥24	1.1 (0.6-2.1)	.66	0.9 (0.6-1.3)	.49	1.5 (1.1-2.1)	.02
**University**							
	Busitema University	1.1 (0.3-3.6)	.88	1.2 (0.7-2.2)	.56	1.4 (0.8-2.4)	.25
	Gulu University	0.3 (0.1-1)	.05	1.5 (0.7-3.1)	.29	1 (0.5-1.9)	.98
	Islamic University in Uganda	0.4 (0.2-1.1)	.07	1.3 (0.7-2.3)	.38	1.7 (1-2.8)	.06
	Kabale University	0.5 (0.2-1.3)	.14	2.1 (1.1-4.1)	.03	2.2 (1.3-3.9)	.01
	Kampala International University	0.6 (0.2-1.9)	.40	1.9 (0.9-3.6)	.07	2.4 (1.3-4.5)	<.001
	King Caesar University	0.7 (0.1-3.5)	.64	2.1 (0.7-6.1)	.16	2.3 (1-5.4)	.06
	Makerere University	1	N/A	1	N/A	1	N/A
	Mbarara University of Science and Technology	0.5 (0.2-1.6)	.25	1 (0.5-1.9)	>.99	1.4 (0.8-2.6)	.25
	Soroti University	2.8 (0.3-27.4)	.38	1.3 (0.4-4.4)	.72	1.6 (0.5-5.1)	.41
**Program**							
	Bachelor of Medicine and Bachelor of Surgery	1	N/A	1	N/A	1	N/A
	Bachelor of Dental Surgery	0.3 (0.1-1.3)	.10	0.5 (0.2-1.2)	.12	0.9 (0.4-2.4)	.87
	Bachelor of Nursing	0.5 (0.2-1.1)	.07	0.7 (0.4-1.3)	.25	1.2 (0.7-2.1)	.57
	Bachelor of Pharmacy	0.3 (0.1-1.1)	.06	0.7 (0.3-1.6)	.35	2.9 (1.2-7.1)	.02
**Year of study**							
	1st	1	N/A	1	N/A	1	N/A
	2nd	1.4 (0.7-3)	.39	0.9 (0.5-1.8)	.86	1 (0.6-1.7)	>.99
	3rd	1.5 (0.7-3.4)	.33	0.8 (0.4-1.6)	.60	1 (0.5-1.7)	.91
	4th	4.1 (1.6-10.3)	<.001	1 (0.5-1.9)	.96	0.8 (0.5-1.5)	.55
	5th	2.5 (0.8-8)	.12	0.7 (0.3-1.5)	.38	1 (0.5-2)	.94
**Source of information on COVID-19^c^**							
	Webinar	0.7 (0.3-1.6)	.44	0.8 (0.5-1.4)	.50	1.2 (0.8-2)	.37
	TV or radio	1.1 (0.6-2.1)	.67	1.7 (1.1-2.6)	.01	0.9 (0.6-1.3)	.47
	Journal and articles	1.3 (0.7-2.5)	.36	1.3 (0.9-1.9)	.20	1.4 (1-2)	.06
	Social media	1.6 (0.9-2.8)	.13	1.1 (0.7-1.7)	.58	1.1 (0.8-1.6)	.66
	Websites	1.3 (0.7-2.2)	.40	0.8 (0.5-1.1)	.22	1.1 (0.8-1.6)	.44
	Online courses	2.1 (0.7-6.4)	.20	1.2 (0.7-2.1)	.56	1.8 (1.1-3.2)	.03

^a^aOR: adjusted odds ratio.

^b^Not applicable.

^c^COVID-19: coronavirus disease.

### Attitudes of Medical Students on COVID-19

Of the 741 medical students in Uganda, 74% (n=550) had a positive attitude toward COVID-19 prevention. The mean attitude score was 20.8 (SD 3.2; Table 3). Most of the participants agreed that they would go for institutional quarantine if they had contact with patients with COVID-19. A total of 80% (n=592) were willing to participate in the management of patients with COVID-19 when called upon. However, 32% (n=236) of Ugandan medical students were not confident that Uganda would contain the pandemic (Table 5).

**Table 5:** Responses of Ugandan medical students (N=741) to questions on attitude toward COVID-19.

Questions	Strongly disagree, n (%)	Disagree, n (%)	Neutral, n (%)	Agree, n (%)	Strongly agree, n (%)
Frequently washing my hands using soap or alcohol-based sanitizers can prevent me from getting COVID-19^a^	32 (4)	6 (1)	4 (1)	253 (34)	446 (60)
Wearing a facemask can protect me from getting COVID-19 infection	20 (3)	42 (6)	24 (3)	441 (60)	214 (29)
I will go into institutional quarantine if I come into contact with a patient with COVID-19	27 (4)	18 (2)	23 (3)	252 (34)	421 (57)
When called upon, I will willingly participate in the frontline of COVID-19 pandemic response	29 (4)	26 (4)	94 (13)	250 (34)	342 (46)
Uganda is in a good position to contain COVID-19 pandemic	39 (5)	74 (10)	123 (17)	322 (43)	183 (25)

^a^COVID-19: coronavirus disease.

On bivariate analysis, sex (*P*=.04), academic program (*P*=.04), and mass media like television and radios (*P*=.01) significantly affected attitudes of medical students on COVID-19 prevention (Table 3). After adjusting the effects of independent variables on attitudes, medical students from Kabale University were 2 times more likely to have a better attitude compared to Makerere University (aOR 2.1, 95% CI 1.1-4.1; *P*=.03; Table 4). Those who obtained information on COVID-19 using mass media (television and radios) were twice more likely to have a positive attitude than their counterparts who used other sources (aOR 1.7, 95% CI 1.1-2.6; *P*=.01; Table 4). Female medical students also significantly had more negative attitudes (aOR 0.7, 95% CI 0.5-1.0; *P*=.04) toward COVD-19 prevention than male students (Table 4).

### COVID-19 Prevention Practices Among Medical Students

Of the 741 students, only 57% (n=426) had good practices toward the prevention of COVID-19. The mean practice score was 11.8 (SD 1.9) indicating moderately good practices (Table 3). The majority of the students had maintained a social distance, refrained from shaking hands, and washed hands before touching their face (Table 6). It is notable that over four-fifths of the medical students had engaged in health education aimed at improving the public’s understanding of COVID-19 (Table 6). Older medical students (aOR 1.5, 95% CI 1.1-2.1; *P*=.02), pharmacy students (aOR 2.9, 95% CI 1.2-7.1; *P*=.02), and KU (aOR 2.2, 95% CI 1.3-3.9; *P*=.01), and KIU (aOR 2.4, 95% CI 1.3-4.5; *P*<.001) students all significantly had better practices compared to students younger than 24 years, MBChB students, and Makerere University medical students, respectively (Table 4). Students who took online courses on COVID-19 also significantly had better practices than others on multivariate analysis (Table 4).

**Table 6:** Responses of Ugandan medical students (N=741) to questions on practices toward COVID-19.

Questions	Always, n (%)	Occasional, n (%)	Never, n (%)
In recent days, I have maintained a social distance of 1 meter with anyone coughing or sneezing	449 (61)	260 (35)	32 (4)
In recent days, I have worn a mask when getting outside home	170 (23)	285 (38)	286 (39)
In recent days, I have refrained from shaking hands	631 (85)	96 (13)	14 (2)
In recent days, I have washed my hands before touching my face	359 (48)	354 (48)	28 (4)
In recent days, I have engaged in health information campaigns on COVID-19^a^	235 (32)	367 (50)	139 (19)

^a^COVID-19: coronavirus disease.

## Discussion

The rapid spread of the COVID-19 pandemic has greatly impacted public health and significantly strained health care systems, especially the medical workers [15]. COVID-19 has also impaired the training of medical students across the world as a result of the closure of schools during the lockdown.

This study sought to determine the perspective of medical students in Uganda toward the COVID-19 pandemic. To the best of our knowledge, this is the first study in Uganda and Africa at-large to examine the perspective toward COVID-19 among health sciences students.

We found that at least 9 in 10 of the medical students had sufficient knowledge, irrespective of their age, sex, university of study, and the course they were pursuing. This level of knowledge is higher than that demonstrated among Iranian medical students (86.96%) [11], Indian health care professionals, students and nonmedical health staff (71%) [16], and Bangladesh students (10.5%) [17]. The study program (MBChB, BDS, BPHARM, and BNUR) and university did not significantly affect knowledge on COVID-19. Fourth year students were 4 times more likely to have good knowledge compared to first year counterparts. Fourth year students in all medical schools in Uganda have at least undergone a junior clerkship in medical wards and have better understanding of disease aspects. This puts them and other medical students who have experienced ward rotations at a good position to participate in the management of patients with COVID-19 once the need arises. However, this cannot explain why they had better knowledge compared to their senior colleagues in 5th year. Perhaps this could be due to the fact that 4th years constituted up to nearly one-third of the study population. Although the majority of the students used mass media to obtain their information, those who used journals or articles and websites significantly had sufficient knowledge more than others. This demonstrates that journal articles and websites are comparatively better reserves for medical knowledge on COVID-19. Peer-reviewed journal articles have been the main stay for dissemination of up-to-date and credible scientific information regarding all aspects of COVID-19. Furthermore, social media although convenient and widely preferred especially by youth, may have a lot of other false content and is not the best portal to relay medical knowledge to students. However, its wide use could be leveraged to convey messages especially on preventive health measures to the public.

In our study, 74% of all participants had positive attitudes compared to 65.4% of participants in a similar Pakistani study [18] who had positive attitudes. However, students who watched TV or heard radio talk shows were 10% more likely to have a good attitude. These sources provide more censored information given that their operations are binding to regulatory guidelines from government agencies like Uganda Communications Commission compared to sources like social media and websites that are less regulated and have had an onslaught of conspiracy theories and misinformation that can ably bias one’s picture of the pandemic. Academic program significantly affected attitude; MBChB students had the most positive attitude probably because they act as frontline health workers and directly interact with patients in most regional referral teaching hospitals during their clinical years on a routine basis. However, this finding could be biased by the disproportionate representation among respondents from different medical courses.

With regard to practices, Iranian medical students had a high rate of preventive practice behaviors compared to their Ugandan counterparts (95% vs 57%). This could be due to recruitment of more senior students in their clinical years (5th to 7th year medical students) in the Iran study compared to our study where we enrolled 1st to 5th year medical students. KU and KIU students were twice more likely to have better practices than MUK students. These two medical schools are all located in Western Uganda. Western Uganda is known for outbreaks of viral hemorrhagic fevers hence priming the health care professionals and trainees on the heightened need for appropriate preventive practices. Pharmacy students also significantly had better practices than MBChB students calling for increased sensitization. Of interest, we also found that over 80% of the medical students in Uganda were willing to participate in frontline care response to COVID-19 if called upon. This finding, combined with the fact that over 80% of the medical students had already engaged in health education aimed at improving the public’s understanding of COVID-19 despite being in lockdown, underscores the enthusiasm medical students have toward providing health services and would effortlessly engage in frontline care if the situation warranted. This is in consonance with a study that reported great willingness by medicine, nursing, and pharmacy students to work during infectious disease outbreaks despite their fears [19]. The health ministry in India also proposed provisional permission of medical undergraduates of senior grades to treat patients with COVID-19 [16]. Therefore, we have a generation of enthusiastic future health care professionals and there is surely widespread consensus that they can play an active role in the pandemic.

Medical students who may wish to join hospital teams managing the COVID-19 outbreaks have a high risk of exposure to the infection given their limited clinical experience. It has been shown among medical students that having and enhancing knowledge about a new infectious disease by fostering cooperation between hospitals and universities will help improve the students’ perceptions of the disease and preventive behaviors [20]. The risk of medical students acquiring coronavirus infection due to lack of enough knowledge about COVID-19 is increased by the fact that there is asymptomatic carrier transmission of the coronavirus, which has been reported [21,22].

The limitation of our study lies on the nonavailability of a validated KAP assessment tool among this population. Sending daily reminders to the eligible participants on the targeted WhatsApp groups lessened possible response bias associated with online surveys. Sampling bias due to convenience sampling used in the study limits the representativeness of the study. However, the relatively large sample size reduces the effect of sampling bias. The study also involved nearly all medical schools across the country.

In conclusion, we were able to demonstrate that Ugandan medical students have sufficient knowledge on COVID-19 and the majority are willing to join the frontline health care response when called upon. Therefore, in the event of escalation in COVID-19 cases in Uganda, medical students, especially those in the clinical years, may be harnessed to work alongside qualified health care professionals in the COVID-19 response. Continued access to online health information resources like free courses, clinical management guidelines, and webinars on COVID-19 offered internationally (eg, by the International Federation of Medical Students Association [23], the CDC [24], and the WHO [25,26]) and nationally (eg, by Ministry of Health-Uganda [27]) may help improve knowledge, attitude, and practices among medical students.

## Appendix

Multimedia Appendix 1 Data collection questionnaire.

## Abbreviations

aOR: adjusted odds ratio
BDS: Bachelor of Dental Surgery
BNUR: Bachelor of Nursing
BPHARM: Bachelor of Pharmacy
BU: Busitema University
COVID-19: coronavirus disease
GU: Gulu University
KAP: knowledge, attitude, and practices
KIU: Kampala International University
KU: Kabale University
Mak: Makerere University
MBChB: Bachelor of Medicine and Bachelor of Surgery
MUST: Mbarara University of Science and Technology
SU: Soroti University
UCU: Uganda Christian University
WHO: World Health Organization
