# Supplementary material for: Perspective of Medical Students on the COVID-19 Pandemic: Survey of Nine Medical Schools in Uganda
Source: JMIR Public Health Surveill. 2020 Jun 19;6(2):e19847. doi: 10.2196/19847 (PMC7307324; doi:10.2196/19847)
Supplement: Multimedia Appendix 1 [file publichealth_v6i2e19847_app1.pdf]

COVID-KAP-Medical-Students Study

KNOWLEDGE, ATTITUDES AND PRACTICES OF MEDICAL STUDENTS IN UGANDA TOWARDS COVID-19.

*Required

Investigators

RONALD Olum (1*)
GAUDENCIA Chekwech (1)
GODFREY Wekha (1)
Dianah R Nassozi (1)
Jonathan Kajjimu (4)
Juliet Kemigisa (5)
Andrew M Kanyike (6)
Paul Mulyamboga (7)
Oscar K Muhoozi (8)
Lauryn E Nsenga (9)
Lyavala Musilim (10)
Asiimwe Asaph (11)
Felix Bongomin (2,3*)

1. College of Health Sciences, Makerere University.
2. Department of Internal Medicine, Makerere University.
3. Department of Medical Microbiology and Immunology, Gulu University.
4. Faculty of Medicine, Mbarara University of Science and Technology.
5. Faculty of Medicine, Gulu University.
6. Faculty of Health Sciences, Busitema University.
7. Faculty of Biology, Medicine and Heallth, King Caeser University..
8. Faculty of Clinical Medicine and Dentistry, Kampala International University.
9. School of Medicine, Kabale University.
10. Faculty of Medicine, Islamic University in Uganda.
11. School of Medicine and Health Sciences, Soroti University

CLICK NEXT TO CONTINUE.

Consent Form

Coronavirus Disease COVID-19 is an emerging global health pandemic that has infected over 1.8 million people and claimed over 100,000 lives globally. The purpose of the study is to assess the knowledge, attitude and practices of medical students in Uganda towards COVID-19. There is no direct benefit of the study to the participants however data from this study shall inform healthcare stakeholders on implementation of outbreak management strategies and preparedness.
Your personal details like emails, name, student's number, registration number and address are not required anywhere in this form.
Participating in the study is voluntary.
In case of any questions related to the study, please contact the Principal Investigator;

Ronald Olum
Email: olum.ronald@gmail.com
Tel: +256775512540

For any queries related to the study participant’s rights, kindly address them to the Chairperson Mulago Hospital Research Ethics Committee,
Dr Frederick Nakwagala
Mobile: +256772325869
A detailed consent form can be downloaded from the link below;

https://drive.google.com/open?id=1j-zvJNxsOu0jOI-FNZXLFe6fSq4WnqF_

Statement of Consent

I have been asked to participate in the research study. The study has been explained to me. I understand what the study means to me including what I (the participant) have to go through while in the study. I have had an opportunity to ask questions about the study and have been answered in the best way for me to understand. If there are any other questions that I have to ask later, I will freely approach the study representatives whose contact I have been provided with. I also understand that my participation is voluntary and my consent can be withdrawn any time I wish to do so, without any penalty or loss of benefits to which I am otherwise entitled to.

1. Tick the box to confirm your participation and click next to continue. *

Tick all that apply.

- [ ] I consent to participate in the study.

Demographics

2. Sex *

Mark only one oval.

- ( ) Female
- ( ) Male

3. Age in completed years *

____

4. University *

Mark only one oval.

- Mbarara University of Science and Technology
- Makerere University
- Kabale University
- Islamic University in Uganda
- Soroti University
- Busitema University
- King Ceasar University
- Kampala International University
- Gulu University

5. Program *

Mark only one oval.

- Bachelor of Pharmacy
- Bachelor of Nursing
- Bachelor of Medicine and Bachelor of Surgery
- Bachelor of Dental Surgery

6. Year of study *

Mark only one oval.

- ( ) 1
- ( ) 2
- ( ) 3
- ( ) 4
- ( ) 5

7. What sources do you frequently use to obtain information on COVID-19. Tick all that apply. *

Tick all that apply.

- [ ] Webinars (online seminars) and conferences
- [ ] Mass Media e.g. TVs, radios, Magazines, Newspapers
- [ ] Journals and articles
- [ ] Social Media e.g. WhatsApp, Facebook, Twitter, Instagram
- [ ] Websites
- [ ] Online Courses

Knowledge

8. SARS-COV-2, the virus that causes COVID-19 is a DNA virus. *

Mark only one oval.

- True
- False

9. The main clinical symptoms of COVID-19 are; (Select the most appropriate features). *

Tick all that apply.

- [ ] Fever
- [ ] Headache
- [ ] Myalgia (muscle pain)
- [ ] Sore throat
- [ ] Runny nose
- [ ] Sneezing
- [ ] Diarrhoea
- [ ] Cough
- [ ] Confusion
- [ ] Difficulty in breathing

10. There is currently no effective cure for COVID-19, but early symptomatic and supportive treatment can help most patients recover from the infection. *

Mark only one oval.

- True
- False

11. Not all persons with COVID-19 will develop severe cases. Only those who are elderly, have chronic illnesses, and are obese are more likely to be severe cases. *

Mark only one oval.

- True
- False

12. Persons with COVID-19 cannot transmit the virus to others when a fever is not present. *

Mark only one oval.

- True
- False

13. The COVID-19 virus spreads via respiratory droplets of infected individuals. *

Mark only one oval.

- True
- False

14. SARS-COV-2 that causes COVID-19 cannot persist on surfaces of objects for hours or days. *

Mark only one oval.

- True
- False

15. Wearing medical masks can prevent one from acquiring infection by the COVID-19 virus. *

Mark only one oval.

- True
- False

16. It is not necessary for children and young adults to take measures to prevent the infection by the COVID-19 virus. *

Mark only one oval.

- True
- False

17. To prevent the infection by COVID-19, individuals should avoid going to crowded places such as bus parks and avoid taking public transportations. *

Mark only one oval.

- True
- False

18. Isolation and treatment of people who are infected with the COVID-19 virus are effective ways to reduce the spread of the virus. *

Mark only one oval.

- True
- False

19. People who have contact with someone infected with the COVID-19 virus should be immediately isolated in a proper place. In general, the observation period is 14 days. *

Mark only one oval.

- True
- False

Attitude

20. Frequently washing my hands using soap or alcohol-based sanitizers can prevent me from getting COVID-19. *

Mark only one oval.

- Strongly disagree
- Disagree
- Not sure
- Agree
- Strongly agree

21. Wearing a face mask can protect me from getting COVID-19 infection. *

Mark only one oval.

- Strongly disagree
- Disagree
- Not sure
- Agree
- Strongly agree

22. I will go into institutional quarantine if I come into contact with a patient with COVID-19. *

Mark only one oval.

- Strongly disagree
- Disagree
- Not sure
- Agree
- Strongly agree

23. When called upon, I will willingly participate in the front-line of COVID-19 pandemic response. *

Mark only one oval.

- Strongly disagree
- Disagree
- Not sure
- Agree
- Strongly agree

24. Uganda is in a good position to contain COVID-19 pandemic. *

Mark only one oval.

- Strongly disagree
- Disagree
- Not sure
- Agree
- Strongly agree

Practices

25. In recent days, I have maintained a social distance of 1 meter with anyone coughing or sneezing *

Mark only one oval.

- Never
- Sometimes
- Aways

26. In recent days, I have worn a mask when getting outside home. *

Mark only one oval.

- Never
- Sometimes
- Always

27. In the recent days, I have refrained from shaking hands. *

Mark only one oval.

- Never
- Sometimes
- Always

28. In the recent days, I have washed my hands before touching my face? *

Mark only one oval.

- Never
- Sometimes
- Always

29. In the recent days, I have engaged in health information campaigns on COVID-19 *

Mark only one oval.

- Never
- Sometimes
- Always

This content is neither created nor endorsed by Google.

Google Forms
